# Supplementary material for: Activity of Amphotericin B-Loaded Chitosan Nanoparticles against Experimental Cutaneous Leishmaniasis
Source: Molecules. 2020 Sep 2;25(17):4002. doi: 10.3390/molecules25174002 (PMC7504813; doi:10.3390/molecules25174002)
Supplement: Supplementary file 1 [file molecules-25-04002-s001.pdf]

**Methods:**

*TEM and SEM*

The morphology of the nanoparticles was examined using a transmission electron microscopy (TEM) at UCL, School of Pharmacy.

Liquid samples for TEM were dropped with a Pasteur pipette onto a copper grid coated with a carbon/formvar support film. After 15 s, a filter paper was blotted off to remove the excess sample. Then a drop of negative stain (1% uranyl acetate) was added and blotted after 15 s. The grid was placed into a specimen holder and inserted into a Phillips/FEI CM 120 BioTwin TEM for imaging at 200 kV.

For the SEM, a sample of nanoparticles was placed onto a self-adhesive carbon disc mounted on a 25 mm aluminium stub. The stub was coated with 25 nm of gold using a sputter coater and placed into a FEI Quanta 200 FEG SEM for imaging at 5 kV accelerating voltage using secondary electron detection.

*Fluorescence Microscopy of Skin Sections Post Formulation Application*

To visualise the nanoparticles, formulations with rhodamine-labelled chitosan were prepared in a similar manner to unlabelled particles and then were characterised regarding size and zeta-potential using the Zeta-sizer and applied to infected and uninfected mouse skin using FDC (blank rhodamine-labelled chitosan-TPP nanoparticles equivalent to 3.93 ± SD mg of AmB/mL loaded in AmB loaded chitosan TPP nanoparticles and blank rhodamine-labelled chitosan-dextran sulphate nanoparticles equivalent to 3.84 ± SD mg of AmB/mL loaded in AmB loaded chitosan TPP nanoparticles) as described above. After the experiment, the cells were dismantled and skin tissue fixed in tris-zinc fixative overnight as described by Accart et al. (2014) (65). After 24 h the skin samples were embedded in gelatin and immersed in OCT before storage at −80°C. Cryosections of 5 μm were cut using a cryostat (Leica CM1950).

For immunohistochemistry, the sections were defrosted and submerged in PBS (37°C) for 30 min to dissolve the gelatine after which they were submerged in PBS for 5 min, counterstained with DAPI and mounted in Prolong Gold (Thermofisher Scientific). Sections were examined using a Zeiss Axio Scan Z1 with a × 20 objective.

**Results:**

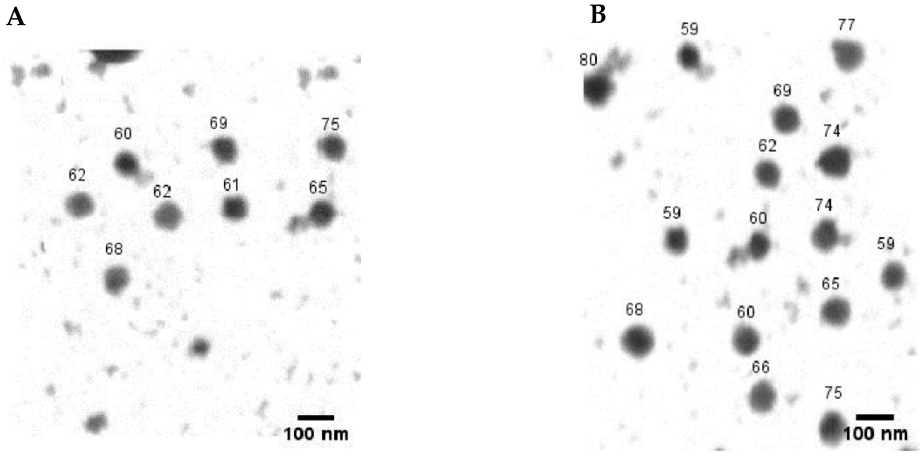

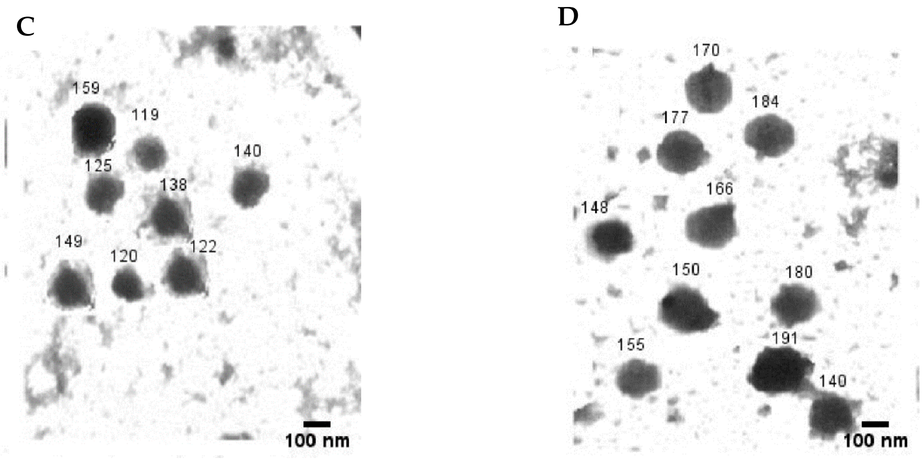


**Figure S1.** TEM micrographs of unloaded and AmB-loaded chitosan nanoparticles. A: Unloaded chitosan–TPP nanoparticles, B: AmB-loaded chitosan–TPP nanoparticles, C: Unloaded chitosan – dextran sulphate nanoparticles, D: AmB-loaded chitosan–dextran sulphate nanoparticles. TEM images indicate the nanoparticles to be spherical. Magnification: 40000×.

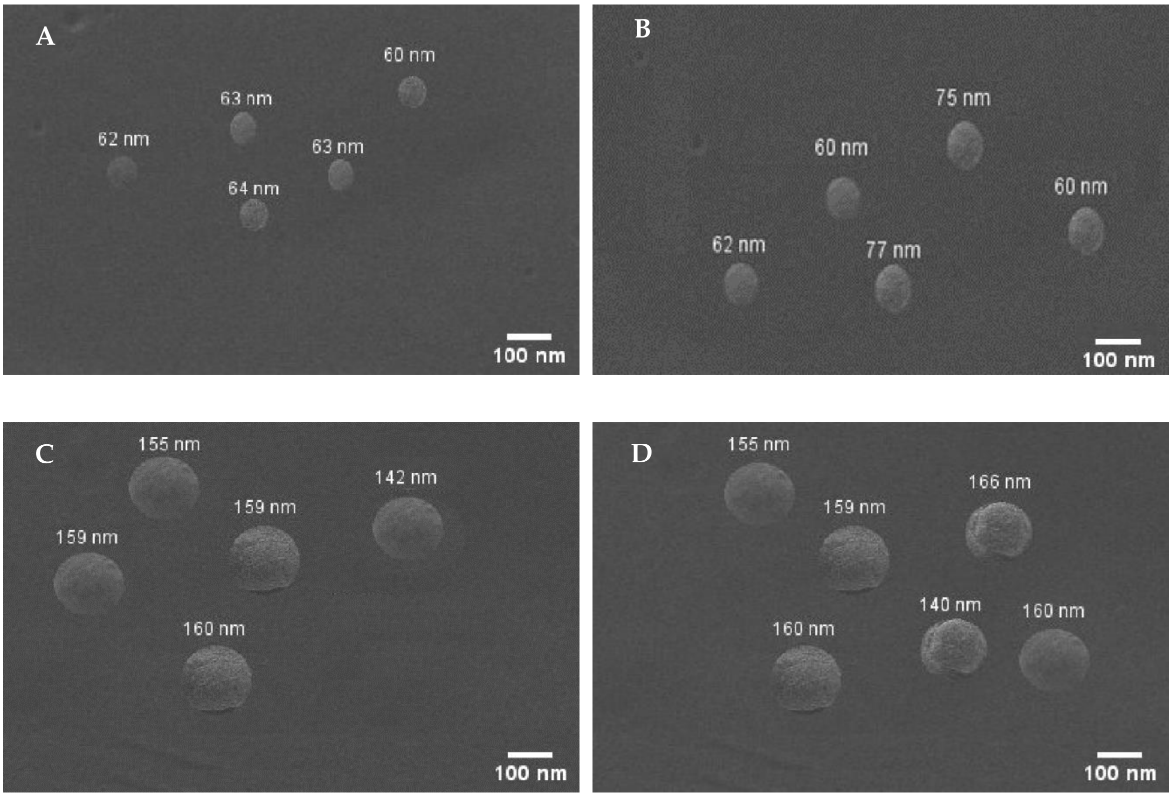


**Figure S2.** SEM micrographs of lyophilised unloaded and AmB-loaded chitosan nanoparticles. A: CH-TPP, B: AmB-CH-TPP, C: CH-Dex, D: AmB-CH-Dex nanoparticles. SEM images indicate the nanoparticles to be spherical and with similar sizes measured by DLS.

*Stability of chitosan nanoparticles following incubation in different media for one month*

Following incubation of drug-loaded chitosan-TPP and of drug-loaded chitosan-dextran nanoparticles in different media (water, PBS, RPMI (pH 7.5 or 6.5), mouse plasma) at different temperatures (4, 34 and 37 °C) for a period of 30 days, no significant changes in particle size or polydispersity index or in zeta potential were found, which indicated a high stability of these nanoparticles (Tables S1 and S2). From Tables S1 and S2, it can also be seen that the nature of the incubation medium had no influence on the size or surface charge of the particles ($p > 0.05$ by one-way-ANOVA).

**Table S1.** Variations of physicochemical properties of AmB-loaded chitosan-TPP nanoparticles in different media upon storage at different temperatures.

| | Day 0 | | | Day 1 | | | Days 7 | | | Days 30 | | |
|---|---|---|---|---|---|---|---|---|---|---|---|---|
| | **Size nm** | **PDI** | **Zeta Potential mV** | **Size nm** | **PDI** | **Zeta Potential mV** | **Size nm** | **PDI** | **Zeta Potential mV** | **Size nm** | **PDI** | **Zeta Potential mV** |
| water at 4, 34 or 37 °C | 70 ± 6 | 0.1± 0.02 | 25.5 ± 1 | 74 ± 5 | 0.2 ± 0.01 | 23.4 ± 1 | 73 ± 5 | 0.2 ± 0.1 | 24.0 ± 1 | 76 ± 5 | 0.2 ± 0.1 | 23.9 ± 1 |
| PBS at 4, 34 or 37 °C | 73± 5 | 0. ± 0.01 | 23.3 ± 1 | 75 ± 4 | 0.1 ± 0.02 | 22.9 ± 2 | 77 ± 4 | 0.2 ± 0.1 | 22.5 ± 1 | 79 ± 5 | 0.2 ± 0.1 | 21.9 ± 1 |
| RPMI (pH = 7.5) at 4, 34 or 37 °C | 75 ± 6 | 0.2 ± 0.1 | 24.1± 1 | 79 ± 7 | 0.2 ± 0.05 | 22.9 ± 1 | 80 ± 7 | 0.2 ± 0.1 | 22.8 ± 1 | 81± 6 | 0.2± 0.1 | 22.1 ± 1 |
| RPMI (pH = 6.5) at 4, 34 or 37 °C | 68 ± 7 | 0.1 ± 0.01 | 32 ± 6 | 74 ± 5 | 0.2 ± 0.09 | 30 ± 4 | 77 ± 5 | 0.1 ± 0.1 | 29 ± 3 | 77 ± 9 | 0.2± 0.1 | 30 ± 3 |
| plasma at 4 °C | 75 ± 7 | 0.1 ± 0.01 | 29 ± 6 | 77 ± 6 | 0.2 ± 0.03 | 30 ± 4 | 79 ± 8 | 0.2 ± 0.1 | 29 ± 3 | 80 ± 7 | 0.3 ± 0.1 | 29 ± 4 |

data expressed as mean +/- SD (experiment was reproduced three times with confirmed similar data). No significant difference was shown in the size, PDI or zeta potential between two types of the nanoparticles after 30 days storage ($p$ > 0.05 by $t$-test).

**Table S2.** Variations of physicochemical properties of AmB-loaded-chitosan dextran sulphate nanoparticles in different media upon storage at different temperatures.

| | Day 0 | | | Day 1 | | | Days 7 | | | Days 30 | | |
|---|---|---|---|---|---|---|---|---|---|---|---|---|
| | **Size nm** | **PDI** | **Zeta Potential mV** | **Size nm** | **PDI** | **Zeta Potential mV** | **Size nm** | **PDI** | **Zeta Potential mV** | **Size nm** | **PDI** | **Zeta Potential mV** |
| water at 4, 34 or 37 °C | 180 ± 6 | 0.2± 0.1 | −14 ± 5 | 187 ± 5 | 0.2± 0.1 | −16 ± 5 | 186 ± 5 | 0.2± 0.1 | −17 ± 5 | 186 ± 5 | 0.2± 0.1 | −17 ± 5 |
| PBS at 4, 34 or 37 °C | 177 ± 5 | 0.2 ± 0.1 | −15 ± 5 | 178 ± 4 | 0.2 ± 0.1 | −14 ± 5 | 183 ± 4 | 0.2 ± 0.1 | −17 ± 5 | 182 ± 4 | 0.2 ± 0.1 | −17 ± 5 |
| RPMI (pH = 7.5) at 4, 34 or 37 °C | 180 ± 6 | 0.2 ± 0.1 | −20 ± 5 | 183 ± 7 | 0.2 ± 0.1 | −17 ± 5 | 183 ± 7 | 0.2 ± 0.1 | −19 ± 5 | 180 ± 7 | 0.2 ± 0.2 | −19 ± 5 |
| RPMI (pH = 6.5) at 4, 34 or 37 °C | 175 ± 7 | 0.2 ± 0.1 | −11 ± 5 | 178 ±5 | 0.2 ± 0.1 | −14 ± 5 | 177 ± 5 | 0.2 ± 0.1 | −13 ± 5 | 181 ± 5 | 0.2 ± 0.1 | −13 ± 5 |
| plasma at 4 °C | 177 ± 7 | 0.2 ± 0.1 | −15 ± 5 | 179 ±5 | 0.2 ± 0.1 | −17 ± 5 | 181 ± 5 | 0.3 ± 0.1 | −13 ± 5 | 187 ± 6 | 0.2 ± 0.1 | −14 ± 5 |

data expressed as mean +/- SD (experiment was reproduced three times with confirmed similar data). No significant difference was shown in the size, PDI or zeta potential of the nanoparticles after 30 days storage ($p$ >0.05 by $t$-test).

**Table S3.** In vitro cumulative release of AmB from the two formulations at different conditions.

| Type | | | 6 h % | 24 h % | 48 h % | 72 h % | 96 h % | 120 h % | 144 h % | 168 h % |
|---|---|---|---|---|---|---|---|---|---|---|
| AmB-loaded chitosan–dextran sulphate nanoparticles | PBS, pH 7.4 | 4 °C | 0.1 ±0.05 | 1 ± 0.05 | 2.2 ± 0.4 | 5.2 ± 1 | 7.5 ± 2 | 9.5 ± 2 | 11 ± 2 | 15 ± 2 |
| | | 34 ° C | 0.3 ± 0.1 | 2.5 ± 0.2 | 5.2 ± 1 | 8.5 ± 2 | 10 ± 3 | 13.5 ± 2 | 16.4 ± 3 | 20 ± 3 |
| | | 37 ° C | 0.1 ± 0.02 | 2 ± 0.1 | 4.4 ± 1 | 6.9 ± 1 | 9.1 ± 2 | 12.5 ± 3 | 15.5 ± 3 | 18.5 ± 2 |
| | PBS, pH 6.5 | 4 ° C | 0.2 ± 0.02 | 2 ± 0.2 | 3.1 ± 1 | 4.9 ± 1 | 6.9 ± 1 | 8.9 ± 1 | 11.5 ± 2 | 15.9 ± 2 |
| | | 34 ° C | 0.4 ± 0.1 | 4 ± 0.5 | 7.3 ± 2 | 9.2 ± 3 | 13.1 ± 3 | 15 ± 2 | 17.2 ± 4 | 21.2 ± 2 |
| | | 37 ° C | 0.1 ± 0.05 | 2.9 ± 0.4 | 5.4 ± 1 | 7.9 ± 2 | 10.1 ± 2 | 12.2 ± 2 | 16.5 ± 3 | 19.5 ± 3 |
| | PBS, pH 5 | 4 ° C | 0.2 ± 0.05 | 3.5 ± 1 | 9.5 ± 2 | 16.1 ± 4 | 17.2 ± 3 | 20.2 ± 3 | 21.1 ± 4 | 32.2 ± 4 |
| | | 34 ° C | 0.5 ± 0.1 | 7.5 ± 2 | 14.5 ± 3 | 20.9 ± 5 | 23 ± 4 | 24.9 ± 3 | 27.5 ± 4 | 41.9 ± 5 |
| | | 37 ° C | 0.3 ± 0.1 | 6.5 ± 1 | 13.5 ± 3 | 20.1 ± 4 | 21.2 ± 5 | 24.2 ± 3 | 26.1 ± 3 | 38.2 ± 4 |
| | Plasma | 37 ° C | 0.2 ± 0.05 | 4.1 ± 1 | 8.1 ± 1 | 9.2 ± 2 | 10.1 ± 2 | 12 ± 2 | 14.9 ± 2 | 22.9 ± 3 |
| AmB-loaded chitosan –TPP nanoparticles | PBS, pH 7.4 | 4 ° C | 0.5 ± 0.1 | 5.1 ± 1 | 9.2 ± 1 | 11.5 ± 2 | 13.8 ± 2 | 15.9 ± 1 | 18.9 ± 2 | 22.9 ± 3 |
| | | 34 ° C | 1.2 ± 0.3 | 9.9 ± 2 | 15.6 ± 2 | 20.6 ± 3 | 24.5 ± 5 | 26 ± 4 | 28.9 ± 5 | 32.5 ± 2 |
| | | 37 ° C | 1 ±0.2 | 10 ±2 | 14.9 ±3 | 19.5 ±2 | 23.5 ±5 | 24.5 ±3 | 27.5 ±4 | 31.5 ±5 |
| | PBS, pH 6.5 | 4 ° C | 0.3 ± 0.1 | 4.1 ± 1 | 10.2 ± 2 | 12.5 ± 2 | 15.8 ± 5 | 17.9 ± 2 | 19.9 ± 3 | 24.5 ± 3 |
| | | 34 ° C | 1.5 ± 0.3 | 10.5 ± 2 | 16.4 ± 4 | 21.9 ± 4 | 26.3 ± 5 | 27.8 ± 3 | 29.8 ± 5 | 32.5 ± 3 |
| | | 37 ° C | 1.2 ± 0.4 | 9.8 ± 1 | 15.2 ± 3 | 20.2 ± 3 | 24.1 ± 5 | 25.6 ± 4 | 28 ± 4 | 32.6 ± 2 |
| | PBS, pH 5 | 4 ° C | 0.9 ± 0.2 | 16.5 ± 3 | 19.8 ± 3 | 25.5 ± 4 | 26.2 ± 4 | 34.5 ± 4 | 40.2 ± 6 | 47.5 ± 4 |
| | | 34 ° C | 1.5 ± 0.4 | 21.2 ± 4 | 27.2 ± 5 | 31.2 ± 3 | 34.6 ± 6 | 39.8 ± 5 | 41.9 ± 5 | 50.8 ± 6 |
| | | 37 ° C | 1.7 ± 0.4 | 20.2 ± 3 | 26.5 ± 6 | 30.2 ± 4 | 33.1 ± 4 | 40.2 ± 5 | 45.2 ± 5 | 51.2 ± 6 |
| | Plasma | 37 ° C | 1.7 ± 0.3 | 11.2 ± 2 | 14.5 ± 4 | 20.9 ± 2 | 25.3 ± 3 | 27.3 ± 4 | 29.9 ± 4 | 33.6 ± 5 |
| AmB solution | PBS, pH 7.4 | 4 ° C | 84 ± 2 | 100 ± 1 | 0 | 0 | 0 | 0 | 0 | 0 |
| | | 34 ° C | 85 ± 2 | 100 ± 2 | 0 | 0 | 0 | 0 | 0 | 0 |
| | | 37 ° C | 86 ± 3 | 100 ± 2 | 0 | 0 | 0 | 0 | 0 | 0 |
| | PBS, pH 6.5 | 4 ° C | 83 ± 1 | 100 ± 1 | 0 | 0 | 0 | 0 | 0 | 0 |
| | | 34 ° C | 86 ± 2 | 100 ± 3 | 0 | 0 | 0 | 0 | 0 | 0 |
| | | 37 ° C | 88 ± 4 | 100 ± 2 | 0 | 0 | 0 | 0 | 0 | 0 |
| | PBS, pH 5 | 4 ° C | 84 ± 1 | 100 ± 2 | 0 | 0 | 0 | 0 | 0 | 0 |
| | | 34 ° C | 85 ± 1 | 100 ± 2 | 0 | 0 | 0 | 0 | 0 | 0 |
| | | 37 ° C | 87 ± 2 | 100 ± 2 | 0 | 0 | 0 | 0 | 0 | 0 |
| | Plasma | 37 ° C | 85 ± 2 | 100 ± 2 | 0 | 0 | 0 | 0 | 0 | 0 |

Data expressed as mean +/- SD (experiment was reproduced three times with confirmed similar data). Both types of nanoparticles showed significantly more cumulative release in the low pH of 5 than in higher pH of 6.5 or 7.5($p < 0.05$ by *t*-test). The AmB release from chitosan-TPP nanoparticles was faster than chitosan dextran sulphate nanoparticles ($p < 0.05$ by *t*-test). AmB-loaded chitosan-TPP nanoparticles size= 69 ± 8 nm and AmB-loaded chitosan-dextran sulphate nanoparticles size= 174 ± 8 nm.

*Haemolysis and cytotoxicity activity of chitosan nanoparticles*

Cytotoxicity ($CT_{50}$ and $CT_{90}$) values of blank and drug-loaded chitosan nanoparticles and for the controls showed that the pH did not influence the cytotoxicity of either formulation ($p > 0.05$, *t*-test-Table 2). Chitosan solution and blank chitosan nanoparticles were the least toxic to red blood cells (RBC) and to KB cells, with the $CT_{50}$ and $CT_{90}$ values of CH-TPP and CH-Dex nanoparticles being similar to each other. Loading the chitosan nanoparticles with AmB increased their toxicity to human RBC and human KB-cells by approximately 3× ($p < 0.05$ by extra sum-of-squares F test), although

**Table S4.** In vitro cytotoxicity of chitosan formulations against Red Blood Cells and KB cells.

| Compound | Properties | $RBC_{50}$ | $RBC_{90}$ | **KB cells pH = 7.5** | | KB Cells pH = 6.5 | |
|---|---|---|---|---|---|---|---|
| | | | | **$CT_{50}$** | **$CT_{90}$** | $CT_{50}$ | $CT_{90}$ |
| | | | | **µg/mL** | | | |
| podophyllotoxin | | | | **0.7 ± 0.03** | **2 ± 0.3** | 0.8 ± 0.04 | 2 ± 0.4 |
| Amphotericin B (AmB solution) | Purity ≥95%, $M_W$ 924.1 | 11.3± 2 | 40.88 ± 5 | 59 ± 2 | 228 ± 2 | 60 ± 2 | 225 ± 3 |
| AmBisome® | Liposomal AmB, Size= 70–80 nm | 525.8 ± 6 | 1782 ± 8 | 401 ± 2 | 1568 ± 2 | 401 ± 3 | 1568 ± 2 |
| HMW chitosan | $M_W$ = 310–375 KDa | 810.1 ± 7 | 3367 ± 9 | 894 ± 4 | 2840 ± 3 | 825 ± 2 | 2864 ± 2 |
| CH-TPP nanoparticles | Size= 67 ± 7 nm, Zeta potential= 28.5 ±1.9 mV | 623.7 ± 6 | 3639 ± 10 | 728 ± 2 | 2858 ± 4 | 696 ± 3 | 2588 ± 4 |
| AmB-CH-TPP nanoparticles | Size= 69 ± 8 nm, Zeta potential= 25.5 ± 1 mV | 209.5 ± 5 | 1129 ± 10 | 356 ± 5 | 1354 ± 5 | 348 ± 3 | 1318 ± 5 |
| CH-Dex nanoparticles | Size= 170 ± 9 nm, Zeta potential= −12.9 ± 3 mV | 621.4 ± 8 | 3341 ± 16 | 949 ± 6 | 2915 ± 6 | 917 ± 2 | 2806 ± 1 |
| AmB-CH-Dex nanoparticles | Size= 174 ± 8 nm, Zeta potential= −11 ± 1 mV | 202.8 ±8 | 931.4 ± 8 | 366 ±3 | 1113 ± 3 | 366 ± 3 | 1131 ±4 |

Experiments were conducted in triplicate, data expressed as mean +/- SD (experiment was reproduced further two times with confirmed similar results; data not shown). A statistically significant difference was found in $RBC_{50}$ (50% haemolytic concentration) values between AmB-loaded chitosan nanoparticles and pure AmB ($p < 0.05$ by using an extra sum-of-squares F test). Blank or AmB-loaded chitosan nanoparticles had a similar toxicity at both pH values (6.5 and 7.5) toward KB-cells ($p > 0.05$ by using *t*-test). A statistically significant difference was found in $CT_{50}$ (50% cytotoxicity dose) values between AmB-loaded chitosan nanoparticles and AmB solution ($p < 0.05$ by using an extra sum-of-squares F test).

**Table S5.** In vitro activity of chitosan formulations and of controls against *Leishmania* promastigotes at pH 6.5 and pH 7.5.

| Compound | Properties | **Medium pH = 7.5 *** | | | | **Medium pH = 6.5*** | | | |
|---|---|---|---|---|---|---|---|---|---|
| | | ***L. major***** | | ***L. mexicana***** | | ***L. major***** | | ***L. mexicana***** | |
| | | $EC_{50}$ µg/mL | $EC_{90}$ µg/mL | $EC_{50}$ µg/mL | $EC_{90}$ µg/mL | $EC_{50}$ µg/mL | $EC_{90}$ µg/mL | $EC_{50}$ µg/mL | $EC_{90}$ µg/mL |
| Amphotericin B (AmB solution) | Purity ≥95%, MW 924.1 | 0.06 ± 0.003 | 0.3 ± 0.02 | 0.2 ± 0.004 | 0.4 ± 0.03 | 0.06 ± 0.003 | 0.3 ± 0.02 | 0.2 ± 0.004 | 0.4 ± 0.03 |
| AmBisome® | Liposomal AmB, Size= 70–80 nm | 1 ± 0.08 | 7 ± 0.3 | 1.8 ± 0.1 | 7 ± 0.07 | 1.1 ± 0.08 | 7 ± 0.1 | 1.9 ± 0.1 | 7 ± 0.01 |
| HMW chitosan | $M_W$ = 310–375 KDa | 106 ± 7 | 539 ± 31 | 141 ± 31 | 556 ± 5 | 7.1 ± 0.5 | 56 ± 4 | 13.5 ± 0.8 | 163 ± 27 |
| CH-TPP nanoparticles | Size= 67 ± 7 nm, Zeta potential= 28.5 ±1.9 mV | 164 ± 6 | 443 ± 10 | 185 ± 10 | 443 ± 0.8 | 28 ±1.5 | 169 ± 11 | 38 ± 0.8 | 173 ± 10 |
| AmB-CH-TPP nanoparticles | Size= 69 ± 8 nm, Zeta potential= 25.5 ± 1 mV | 0.08 ± 0.003 | 0.5 ± 0.02 | 0.3 ± 0.02 | 0.7 ± 0.02 | 0.06 ± 0.003 | 0.4 ± 0.02 | 0.2 ± 0.004 | 0.4 ± 0.02 |
| CH-Dex nanoparticles | Size= 170 ± 9 nm, Zeta potential= −12.9 ± 3 mV | No activity up to 486 | | | | | | | |
| AmB-CH-Dex nanoparticles | Size= 174 ± 8 nm, Zeta potential= −11 ± 1 mV | 0.09 ± 0.003 | 0.4 ± 0.01 | 0.5 ± 0.02 | 1 ± 0.07 | 0.06 ± 0.003 | 0.3 ± 0.02 | 0.4 ± 0.02 | 1.5 ± 0.04 |
| TPP | $M_W$= 367.864 g/mol | No activity up to 486 | | | | | | | |
| dextran sulphate | $M_W$= 40 KDa | No activity up to 486 | | | | | | | |

Experiments were conducted in triplicate cultures, data expressed as mean +/- SD (experiment was reproduced further two times with confirmed similar data not shown). *Statistically significant differences were found for the $EC_{50}$ values of chitosan or CH-TPP at pH = 6.5 and pH = 7.5 ($p < 0.05$ by using *t*-test). ***L. major* promastigotes were significantly more susceptible to AmB solution and AmB-loaded chitosan nanoparticles than *L. mexicana* ($p < 0.05$ by extra sum-of-squares F test). AmB solution, AmB-CH-TPP and AmB-CH-Dex had a similar anti-leishmanial activity.

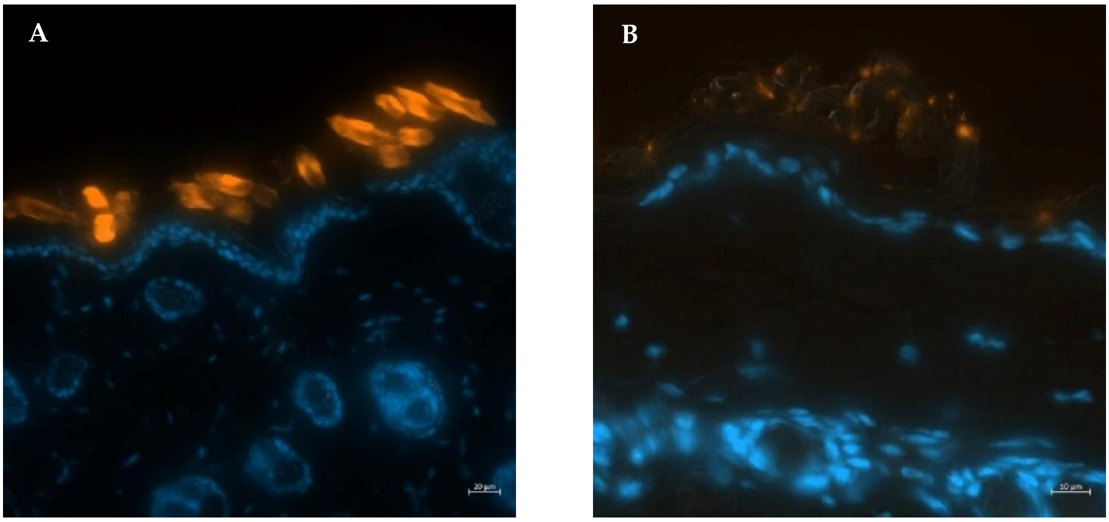


**Figure S3.** Fluorescence images of skin penetration (uninfected and *L. major* infected skin) of blank rhodamine labelled chitosan nanoparticles (**A**) and rhodamine labelled chitosan solution (**B**). We found the same scene for both types of nanoparticles and in both uninfected and infected skin. The red signals (refer to rhodamine labelled chitosan) indicated that the three formulations remained on the surface of skin.
